# Optimizing Sleep Disorder Diagnosis in Underserved Regions: Insights from Video-Polysomnography Analysis in Southern Brazil

**DOI:** 10.1055/s-0046-1825527

**Published:** 2026-07-24

**Authors:** Marco A. S. B. Fleuri, Günther J.L. Gerhardt, Denise M. Zancan, Marino M. Bianchin, Suzana V. Schönwald

**Affiliations:** 1 Medicine School, Universidade Federal do Rio Grande do Sul (UFRGS), Porto Alegre, RS, Brazil; 2 Universidade de Caxias do Sul (UCS), Caxias do Sul, RS, Brazil; 3 Neuroscience Graduate Program, Department of Physiology, Instituto de Ciências Básicas da Saúde (ICBS), Universidade Federal do Rio Grande do Sul (UFRGS), Porto Alegre, RS, Brazil; 4 Neurophysiology Unit, Neurology Division, Hospital de Clínicas de Porto Alegre (HCPA), Porto Alegre, RS, Brazil; 5 Graduate Program in Medical Sciences, Universidade Federal do Rio Grande do Sul (UFRGS), Porto Alegre, RS, Brazil; 6 Center for the Treatment of Refractory Epilepsy (CETER), Hospital de Clínicas de Porto Alegre (HCPA), Porto Alegre, RS, Brazil; 7 Basic Research and Advanced Investigations in Neuroscience (BRAIN) Thematic Laboratory, Centro de Pesquisa Experimental (CPE), Hospital de Clínicas de Porto Alegre (HCPA), Porto Alegre, RS, Brazil

**Keywords:** sleep, video-polysomnography, nocturnal motor behaviors, VPSG, RBD

## Abstract

Video-polysomnography (VPSG) is indicated for cases of nocturnal motor behaviors, particularly rapid eye movement (REM) sleep behavior disorder (RBD), and different sleep complaints in the context of neurological disease, particularly neurodegenerative disease. In a location with limited VPSG availability, referral and poststudy diagnoses were reviewed to help create flow protocols for sleep centers in economically-disadvantaged areas.

A total of 115 VPSG studies conducted at a public university-affiliated hospital in Porto Alegre, Southern Brazil, were divided into groups related to sleep complaints that were primarily motor (M; *n* = 54) or non-motor (NM; *n* = 61). Abnormal motor activity was more common in the M group, suggesting that clinical history was sensitive in identifying motor behaviors. However, while most M group referrals were related to RBD suspicion ( *n* = 43; 79.6%), less than half of these were confirmed ( *n* = 21; 48.8%). There was a high ( *n* = 38; 70.4%) prevalence of obstructive sleep apnea (OSA) in the M group, with 14 (25.9%) subjects fully meeting the criteria for moderate-to-severe OSA. In cases of suspected RBD ( *n* = 43), the rate of moderate-to-severe OSA ( *n* = 20; 46.5%) was nearly the same as the rate of positive RBD diagnosis ( *n* = 21; 48.8%). Prevalence of any severity OSA ( *n* = 35; 81.4%) exceeded positive or incomplete findings for RBD ( *n* = 28; 65.1%). The findings suggest that the suspicion accuracy for OSA and RBD could improve through more thorough history-taking in the prediagnostic workup. In world regions with limited VPSG availability, clinicians must recognize the likelihood of sleep apnea related to nocturnal motor activity. We suggest prioritizing type 1 polysomnography (PSG) to treat or exclude OSA rather than starting an empirical treatment for RBD while waiting for a VPSG study.

## Introduction

Abnormal nocturnal motor activity includes a range of distinct clinical conditions, such as nocturnal epilepsy, rapid eye movement (REM) and non-REM (NREM) sleep-related parasomnia, movement disorders, obstructive sleep apnea (OSA), psychiatry disorders, and even factitious and criminal behaviors. 1 In most cases, a careful clinical evaluation coupled with a laboratory workup, potentially involving a daytime electroencephalogram (EEG) and/or standard sleep study (type-1 polysomnography [PSG]), are sufficient to establish the diagnosis. 2 However, when the diagnosis remains uncertain, when treatment is ineffective, or when there are elements of concern, such as potentially-harmful behavior, a sleep study with audio and video monitoring (video- polysomnography [VPSG]) with extended EEG and/or electromyography (EMG) coverage may be warranted. 3 4 Video/audio monitoring is also recommended as an essential PSG component in the presence of central nervous system (CNS) pathology, particularly in cases of degenerative CNS disease, in which sleep architecture and microstructure may be sufficiently compromised to obscure the electrophysiological characterization of sleep states, and in situations of extreme behavioral-electrophysiological dissociation of sleep/wake states, known as *status dissociatu* s. 5 Video- polysomnography with quantification of motor activity during REM sleep currently represents the gold standard for the diagnosis of REM sleep behavior disorder (RBD). 6 7 The current indications for VPSG may be summarized as: investigation of nocturnal motor behaviors in any clinical context and investigation of any sleep complaint in the context of neurological disease, particularly neurodegenerative disease.

However, VPSG studies, as well as sleep laboratories in general, are not uniformly accessible worldwide. For instance, the Chinese Sleep Research Society 8 reports the presence of more than 3 thousand sleep laboratories in China, a country with a population of approximately 1.4 billion people. In contrast, the Indian Society for Sleep Research 9 recently identified only 22 clinical sleep laboratories in India, which also has a large population of approximately 1.4 billion people. A recent survey 10 of sleep facilities in Africa, with a population of approximately 1.3 billion, found 41 sleep laboratories primarily located in South Africa, Egypt, Nigeria, and Kenya, with most facilities being privately-owned.

The specifications of a full VPSG study go beyond the inclusion of synchronized video in all standard PSG studies, as already recommended in current Brazilian and international guidelines. 7 11 12 Compared with type-1 PSG, VPSG demands greater resources due to the need for additional staff training to recognize abnormal motor activity, the necessity for extra sensors and devices, the requirement for higher-resolution digital amplifiers, and increased computational processing demands. 3 In regions with limited access to sleep centers, investigation protocols may require local adaptation to maximize effectiveness. 4 Such adaptations should consider regional levels of sleep medicine expertise, disease referral patterns, and prevalence of sleep disorders. Unfortunately, beyond reports from major referral centers, there is limited evidence to guide local healthcare providers regarding the demand for VPSG, patient profile, and the diagnostic yield of the procedure. This information is essential to support the development of tailored sleep-healthcare protocols. 13 14

Porto Alegre is the capital of Rio Grande do Sul, the southernmost state of Brazil. It is approximately 800 km equidistant from major cities such as Buenos Aires, in Argentina, São Paulo, in Southeastern Brazil, and Montevideo, in Uruguay. Fewer than two dozen private sleep laboratories are advertised in the local media, with six currently accredited by the Brazilian Sleep Academy (Academia Brasileira do Sono, ABS, in Portuguese). 15 Out of three sleep laboratories currently accredited by the Brazilian Unified Health System (Sistema Único de Saude, SUS, in Portuguese) to serve a state population of nearly 11.5 million inhabitants, only our laboratory performs VPSG studies (16). 16 In local medical schools, the total curricular time dedicated to sleep and sleep disorders is estimated to be around the global average of 2.5 hours, as surveyed over a decade ago. 17 18

In the current study, we have reviewed data from 115 VPSG studies conducted at a public university-affiliated hospital in Porto Alegre to investigate nocturnal motor behaviors and other sleep complaints in the context of neurological disease. We retrospectively analyzed the demographic profile of the patients, the clinical complaints before the sleep studies, and the diagnoses after the sleep studies. Through this research, we aim to contribute to the development of protocols specifically tailored for sleep centers located in economically-disadvantaged regions with limited resources. Maximizing the use of available resources is crucial to improve sleep-health quality in these areas, potentially providing benefits to a larger number of patients in need.

## Materials and Methods

### Sleep Study Selection and Patient Information

**Fig. 1:**
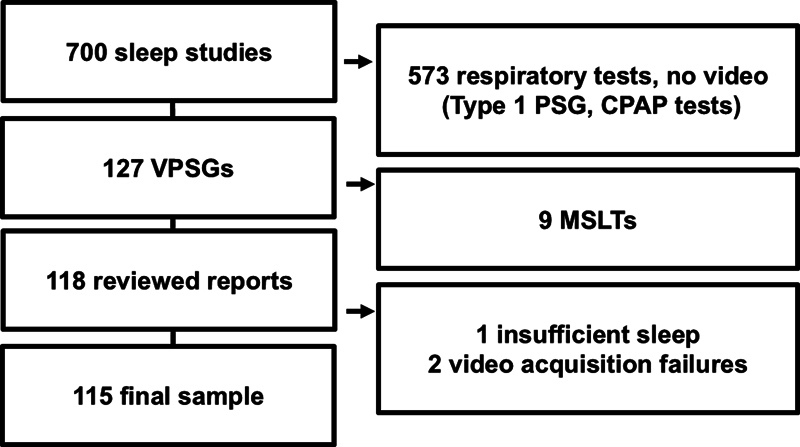
Selection of VPSG reports reviewed in the study. **Abbreviations:** CPAP: continuous positive airway pressure; MSLTs: multiple sleep latency tests; PSG, polysomnography; VPSG, video- polysomnography. **Note:** Type-1 PSG is the standard respiratory polysomnography.

The current study was conducted at the Sleep Laboratory of Hospital de Clínicas de Porto Alegre (HCPA). From a total of 700 sleep studies performed between August 2013 and October 2015, all non-video, type-1 PSGs and continuous positive airway pressure (CPAP) titrating studies, as well as studies conducted prior to a multiple sleep latency test, video acquisition failures, and reports of insufficient sleep were excluded, thus obtaining a final sample of 115 VPSG studies ( Fig. 1 ). The study obtained approval from the institutional review board (HCPA/GPPG Ethics Committee) under protocol number 14–0537. Informed consent was waived by the institutional review board, as this was a retrospective observational study and all data provided was anonymized.

Medical charts and questionnaires administered on the night of the study were carefully reviewed. The data included patient age, sex assigned at birth, body mass index (BMI), subjective sleepiness assessed by the Epworth Sleepiness Scale (ESS), 19 20 OSA symptoms assessed by the Snoring, Tiredness, Observed Apnea, High Blood Pressure, Body Mass Index, Age, Neck Circumference, and Gender (STOP-BANG) screening questionnaire with a threshold score of 3 points, 21 neurological and general chronic disorders, ongoing medication use, and reasons for conducting the VPSG investigation.

The patients were categorized into motor (M) or non-motor (NM) sleep disorder suspicion groups, depending on the VPSG referral reason noted in their medical records. In cases of multiple sleep complaints and based on information from the sleep questionnaires, the potential overlap of diagnostic groups was documented. Patients exhibiting complaints of nocturnal motor activity were assigned to the prestudy M group. An additional subgroup analysis was conducted to explore poststudy OSA and RBD diagnoses in patients with a prestudy suspicion of RBD. Sleep disorders were classified according to the International Classification of Sleep Disorders, Second Edition (ICSD-2). 22

### Sleep Study Description and Sleep Information

Whole-night VPSG studies were conducted by ABS-certified EEG/PSG technicians with a 64-channel digital system from EBNeuro, with 16-bit resolution and sampling rate of 256 Hz. After acquisition and analysis with the Embla Sandman sleep analysis software (Natus Medical Incorporated), the exams were stored in an external hard disk. The recording protocol followed the guidelines of the American Academy of Sleep Medicine (AASM). 23 The patients continued their usual medication regimens, including antiseizure medications (ASMs). Scalp EEG, eye movement, chin and leg EMG, electrocardiogram, snoring, airflow via an oronasal thermistor, airway pressure via a nasal cannula, thoracic and abdominal respiratory effort, body position, and pulse oximetry were collected. Additionally, the upper-limb EMG was recorded from the left and right flexor digitorum superficialis (FDS) muscles to investigate RBD. 24 In the standard VPSG, silver electrodes were placed at eight 10–20 International System EEG positions: F3, C3, O1, A1, F4, C4, O2, and A2. In the EEG-VPSG, 15 additional electrodes were used at the following positions: Fp1, FpZ, Fp2, F7, Fz, F8, Cz, T3, T4, T5, T6, P3, Pz, P4, and Oz. All studies included infrared video and audio monitoring. Each patient participated in a single VPSG session. Sleep stages, arousals, and motor and respiratory events were visually scored by a trained polysomnographer in accordance with standard guidelines, applying the AASM 2012 recommended hypopnea scoring rule. 23

The study reports were retrospectively reviewed to extract information on sleep architecture, arousal index, apnea-hypopnea index (AHI), oxygen desaturation (defined as sleep time in minutes below an oxygen saturation level of 90%), periodic limb movements during sleep (PLMS index), and other motor patterns. Sleep-architecture variables included total sleep time (TST), sleep efficiency, and percentage of time spent in sleep stages N1 + N2, N3, and R. Excessive REM sleep-related motor activity was classified as a categorical variable, indicating the presence or absence of REM sleep without atonia (RWA), as three different criteria to assess REM sleep-related motor activity had been used throughout the study period. For most patients, RWA was assessed qualitatively by simple visual inspection of REM sleep, as recommended in the ICSD-2. 22 Following a transition period when the staff was trained on distinct quantitative assessments of tonic activity in the chin and phasic activity in the limbs, we adopted the Sleep Innsbruck Barcelona (SINBAR) Index, which measures the percentage of 30-second REM epochs with at least five 3-second mini-epochs of “any” (tonic plus phasic) and/or phasic EMG activity in the left or right FDS, with a cutoff threshold of 27%.. 24 Since SINBAR Index was only applied to a minority of patients, the RWA index is not reported. The presence of RWA was only assessed in studies containing at least 15 minutes of unequivocal REM sleep.

Relevant nocturnal motor behaviors documented on video were classified into five categories: 1) REM sleep-related dream enactment behavior (DEB); 2) NREM sleep-related behavior (NRB); 3) wake-related movements suggestive of leg discomfort; 4) ambiguous sleep-related behaviors (arising from ambiguous, non-classifiable sleep and/or related to status dissociatus); and 5) epileptic seizure. More than one type of behavior may be present within the same recording. To assess how VPSG findings contributed to the diagnostic workup, specifically regarding the confirmation of prestudy hypotheses or the addition of new information, relevant video findings were analyzed separately and in conjunction with extended EEG data when available.

### Statistical Analysis

The clinical and sleep profile of the NM and M and the female and male patient groups were compared using the Mann–Whitney U test (MW-U) for the continuous variables. The categorical variables were analyzed with the Chi-squared test or the Fisher's exact test, depending on the sample size. Proportions of RBD and non-RBD diagnoses before and after the VPSG study were compared with the McNemar's exact *p* -value binomial test. A significance level of 0.05 was adopted for all statistical tests. The diagnostic yield of the VPSG study and of the VPSG combined with the extended EEG protocol was analyzed descriptively. Unless otherwise specified, continuous demographic and sleep-architecture variables were reported as mean ± standard deviation values. All analyses were performed using the R statistical computing platform (R Foundation for Statistical Computing).

## Results

### General Clinical Profile and Sleep Architecture

A total of 115 exams pertaining to 54 (47.0%) male and 61 (53.0%) female patients were analyzed. The demographic profile, neurologic diagnoses, and medications used by the sample are shown in Table 1 . Key polysomnographic findings are shown in Table 2 .

**Table 1:** Clinical profile of the pre-study motor (M) and non-motor (NM) groups

Clinical profile	Total sample ( *n* = 115)	M group ( *n* = 54)	NM group ( *n* = 61)	*p* -value ^a^
Male sex: n (%)	54 (47.0)	32 (59.3)	22 (36.0)	< 0.05
Mean age (years)	55.1 ± 16.2	56.5 ± 17.2	53.9 ± 15.4	ns
Age range (years)	11–81	13–81	11–79	
Mean BMI (kg/m ^2^ )	27.5 ± 5.4	27.5 ± 4.9	28.0 ± 5.4	ns
Mean ESS score	10.2 ± 6.0	11.0 ± 4.9	9.4 ± 6.8	ns
Mean STOP-BANG score	3.8 ± 1.6	3.7 ± 1.7	3.9 ± 1.7	ns
Neurological disorders: n (%)				
* Non*	32 (27.8)	11 (20.4)	21 (34.4)	ns
* Tremor/Parkinsonism*	33 (28.7)	26 (48.1)	7 (11.5)	< 0.001
* Epilepsy*	13 (11.3)	5 (9.3)	8 (13.1)	ns
* Neuromuscular*	13 (11.3)	2 (3.7)	11 (18.0)	< 0.05
* Cerebrovascular*	8 (7.0)	4 (7.4)	4 (6.6)	ns
* Hereditary ataxia*	5 (4.3)	3 (5.6)	2 (3.3)	ns
CNS medication use; n (%)				
* None*	27 (23.5)	12 (22.2)	15 (24.6)	ns
* Antidepressants*	55 (47.8)	27 (50.0)	28 (46.0)	ns
* Anti-Parkinson agents*	30 (26.1)	23 (42.6)	7 (11.5)	< 0.001
* ASMs*	28 (24.3)	12 (22.2)	16 (26.2)	ns
* Benzodiazepines/hypnotic drugs/opioids*	12 (10.4)	7 (13.0)	5 (8.2)	ns
* Antipsychotics*	7 (6.1)	5 (9.3)	2 (3.3)	ns
General medication use: n (%)				
* None*	44 (38.3)	14 (26.0)	30 (49.2)	< 0.05
CV risk-related drugs: n (%)				
* Antihypertensives/CV agents*	48 (41.7)	24 (44.4)	24 (39.3)	ns
* Antiplatelet agents*	17 (14.8)	7 (13.0)	10 (16.4)	ns
* Statins*	23 (20.0)	14 (26.0)	9 (14.8)	ns
* Hypoglycemic agents*	12 (10.4)	6 (11.1)	6 (9.8)	ns
Other drugs: n (%)				
* Proton pump inhibitors*	26 (22.6)	11 (20.4)	15 (24.6)	ns
* Thyroid hormones*	10 (8.7)	6 (11.1)	4 (6.6)	ns
* Oral steroids*	8 (7.0)	5 (9.3)	3 (4.9)	ns
* Antiandrogen receptor/Alpha-adrenergic receptor blockers*	6 (5.2)	5 (9.3)	1 (1.6)	ns
* Bronchodilators*	5 (4.3)	3 (5.6)	2 (3.3)	ns

**Abbreviations:** ASMs, antiseizure medications; BMI, Body Mass Index; CNS, central nervous system; CV, cardiovascular; ESS, Epworth Sleepiness Scale; ns, not significant; STOP-BANG, Snoring, Tiredness, Observed Apnea, High Blood Pressure, Body Mass Index, Age, Neck Circumference, and Gender.

**Note:**^a^ The continuous variables were analyzed through the Mann–Whitney U test (MW-U), and the categorical variables, through the Chi-squared test or Fisher's exact test, according to sample size.

**Table 2:** Sleep-architecture and respiratory and motor findings of the primarily motor (M) and non-motor (NM) groups

Sleep profile	Total Sample ( *n* = 115)	M group ( *n* = 54)	NM group ( *n* = 61)	*p* -value ^a^
Sleep architecture				
* Mean TST (minutes)*	344.8 ± 72.5	343.1 ± 67.2	348.7 ± 72.3	ns
* Mean percentage of sleep efficiency*	77.4 ± 15.2	76.7 ± 15.2	78.3 ± 14.2	ns
* Mean percentage of N1* *+* *N2*	65.7 ± 11.5	66.8 ± 11.8	65.6 ± 11.0	ns
* Mean percentage of N3*	21.6 ± 9.7	20.5 ± 8.6	21.8 ± 10.0	ns
* Mean percentage of REM sleep*	12.7 ± 7.6	12.7 ± 7.3	12.5 ± 7.7	ns
* Mean arousal index/hour*	21.2 ± 13.6	19.8 ± 13.5	22.4 ± 14.0	ns
Respiratory findings				
* Mean AHI*	15.8 ± 17.3	13.0 ± 15.2	20.2 ± 18.7	0.01
* AHI ≥ 15: n (%)*	44 (38.3)	14 (25.9)	30 (49.2)	0.01
* AHI ≥ 5: n (%)*	88 (76.5)	38 (70.4)	50 (82.0)	ns
* Mean T90% (minutes)*	7.4 ± 16.1	4.9 ± 11.3	10.2 ± 19.8	ns
Motor findings				
* RWA (n = 102): n (%)*	29 (25.2)	22 (45.8)	7 (13.0)	< 0.001
* Mean PLMS index*	12.2 ± 19.6	12.0 ± 12.9	10.3 ± 21.1	< 0.01
* PLMS ≥ 15: n (%)*	24 (20.9)	14 (25.9)	10 (16.4)	ns

**Abbreviations** : AHI, apnea-hypopnea Index; N1, N2, N3, non-rapid eye movement (NREM) sleep stages 1, 2 and 3; ns, not significant; PLMS, periodic leg movements in sleep/hour; REM, rapid eye movement sleep; RWA, REM sleep without atonia; T90%, oxygen saturation (SpO2) < 90%; TST, total sleep time.

**Note:**^a^ Mann–Whitney U test.

Men ( *n* = 54; 47.0%) and women ( *n* = 61; 53.0%) exhibited similar mean values regarding age (53.7 ± 18.9 and 56.3 ± 13.4 years respectively; W = 1660.5; *p* = 0.94), BMI, (27.7 ± 5.5 and 27.8 ± 4.9 kg/m ^2^ respectively; W = 1626; *p* = 0.90) and ESS scores (10.5 ± 5.3 and 9.86.6 points respectively; W = 1441.5; *p* = 0.48). In total, 71 (61.7%) patients were receiving continuous treatment for clinical conditions. These consisted of heart disease and/or hypertension ( *n* = 48; 41.7%), musculoskeletal disorders ( *n* = 19; 16.5%), diabetes ( *n* = 17; 14.8%), peptic disease ( *n* = 15; 13%), cancer ( *n* = 9; 7.8%), and chronic lung disease ( *n* = 5; 4.3%).

Neurological diagnoses were established in 83 (72.2%) patients. Within this subsample, movement disorders with parkinsonism and/or tremor were the most prevalent category ( *n* = 33; 39.8%): Parkinson disease (PD; *n* = 24; 72.7%); unspecified parkinsonism ( *n* = 6; 18.2%); essential tremor ( *n* = 2; 2.4%) and unclassified tremor ( *n* = 1; 1.2%). Other neurological-disease categories were epilepsy, neuromuscular disorders, cerebrovascular disease, and hereditary ataxia, as described in Table 1 . Additionally, 5 (6.0%) patients had primary headache, while memory or attention disorders were reported in 4 (4.8%) patients, and 1 (1.2%) male patient had undergone surgical treatment for craniopharyngioma. Overall, most patients ( *n* = 88; 76.5%) were prescribed at least one psychoactive medication. Only 1 (0.9%) participant was using melatonin.

Table 1 also shows the profileof the patients in the prestudy M and NM groups. A predominance of male subjects was observed in the M group, while a predominance of female undividuals was noted in the NM group. Consistent with expectations, movement disorders with tremor/parkinsonism and the use of anti-Parkinson agents were more prevalent in the M group, whereas neuromuscular disorders were more frequently reported in the NM group. In both groups, antidepressants represented the most prescribed drug class.

### VPSG Motor and Respiratory Findings in the Prestudy M and NM Groups

Table 2 shows the sleep-architecture findings in the prestudy M and NM groups. Despite the differing reasons for VPSG referral, both groups exhibited similar sleep-architecture characteristics. Additionally, Table 2 shows the motor and respiratory findings of both groups. Information on RWA was available for 102 sleep examinations (48 in the M group and 54 in the NM group). In total, 11 (9.6%) examinations contained less than 15 minutes of unequivocal REM sleep and, in 2 (1.7%) cases, global sleep microstructure was severely disrupted.

The presence of RWA was associated with antidepressant use in 17 (58.6%) cases. Specifically, in the absence of prior motor symptoms (NM group, 7 cases), the presence of RWA was associated with antidepressant use alone (2 cases), antidepressant use combined with a parkinsonian syndrome (4 cases), or a neurodegenerative disease alone (hereditary ataxia, 1 case). In the presence of prior motor symptoms (M group, 22 cases), RWA corresponded to isolated RBD (iRDB; 2 cases), antidepressant-associated RBD (2 cases), neurodegenerative disease-associated RBD (9 cases), and neurodegenerative disease-associated RBD combined with antidepressant use (8 cases). One (4.5%) person with epilepsy belonging to the M group had RWA associated with antidepressant use, in the absence of RBD suspicion.

As expected, the median AHI and the prevalence of moderate-to-severe OSA were higher in the NM group, whereas the prevalence of RWA and the median PLMS index were higher in the M group. However, in the M group, there was a high prevalence of AHI ≥ 5. In total, 24 patients (44.4% of the sample) possibly met the criteria for a diagnosis of mild OSA, contingent upon the presence of additional clinical information. Furthermore, 14 patients (25.9% of the sample) fully satisfied the criteria for moderate-to-severe OSA.

### Diagnostic Yield of the VPSG Procedure

**Fig. 2:**
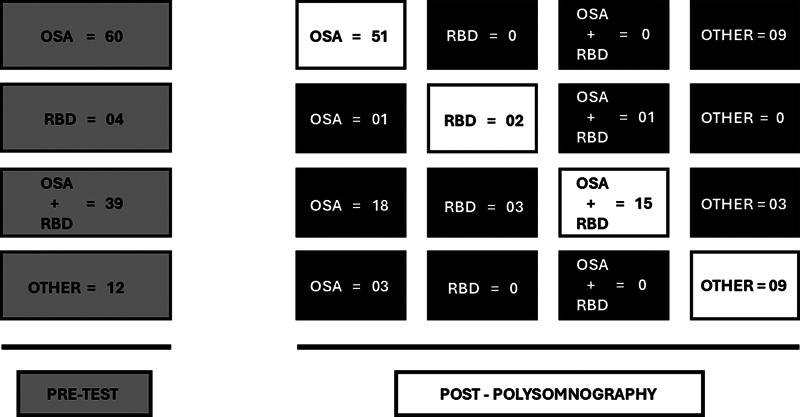
Pre- and postpolysomnography overall diagnostic workup. Less than half of the cases of RBD suspicion were confirmed, whereas most cases of suspicion of OSA and of other diagnoses were corroborated. **Abbreviations:** OSA, obstructive sleep apnea; RBD: rapid eye movement sleep behavior disorder.

Fig. 2 shows how the overall diagnostic workup was affected by the VPSG study. Less than half of the cases of RBD suspicion were confirmed ( *n* = 21; 48.8%), whereas the majority of OSA and other diagnostic suspicions were corroborated. The poststudy diagnoses for the 9 (15.0%) patients with unconfirmed OSA in the absence of RBD suspicion were NREM parasomnia ( *n* = 3; 4.9%), insomnia ( *n* = 3; 4.9%), restless legs syndrome (RLS) with PLMS ( *n* = 2; 3.3%), and epilepsy ( *n* = 1; 1.7%).

**Fig. 3:**
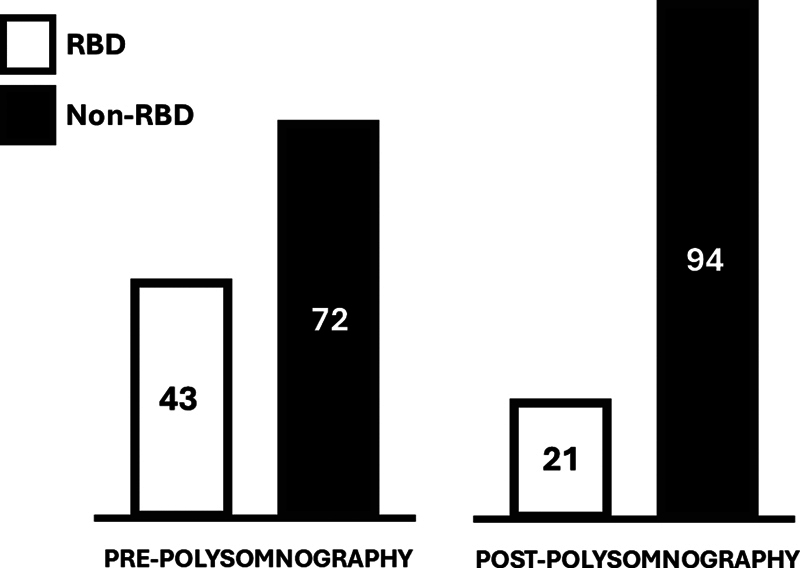
Proportion of RBD versus non-RBD diagnoses before and after the VPSG study (McNemar's exact *p* -value binomial test; *p* < 0.0001).

Fig. 3 shows that the proportions of RBD and non-RBD diagnoses were significantly affected by the VPSG study (McNemar's exact *p* -value binomial test; *p* < 0.0001). Unconfirmed RBD suspicion cases ( *n* = 22; 51.2%) were diagnosed with OSA ( *n* = 19; 96.4%), mild PLMS ( *n* = 2; 9.1%) and status dissociatus with insufficient REM sleep ( *n* = 1; 4.5%). A total of 4 (21.0%) unconfirmed RBD patients diagnosed with OSA had insufficient REM sleep for RWA analysis. In other words, among those with suspected RBD ( *n* = 43), the presence of moderate to severe OSA ( *n* = 20; 46.5%) was nearly as likely as receiving a positive RBD diagnosis ( *n* = 21; 48.8%). The prevalence of any severity OSA ( *n* = 35; 81.4%) exceeded the rate of positive or incomplete findings for RBD ( *n* = 28; 65.1%).

Only 12 patients (10.4%) had a non-RBD, non-OSA prestudy hypothesis. The Poststudy diagnoses in this subsample corresponded to insomnia ( *n* = 5; 41.7%), drug-induced hypersomnolence ( *n* = 2; 16.7%), NREM parasomnia ( *n* = 2; 16.7%), epilepsy ( *n* = 1; 8.3%) and RLS with PLMS ( *n* = 1; 8.3%). Three (25.0%) of these 12 patients also had OSA.

In 28 studies (24.3% of the entire sample), nocturnal motor behaviors that may suggest specific sleep disorders were identified through video analysis. These behaviors comprised DEB in 15 cases (13.0%), NRB in 8 cases (7.0%), waking up with leg discomfort in 3 cases (2.6%) and status dissociatus-related ambiguous behaviors in 2 cases (1.7%). Most of these video findings supported the initial clinical hypotheses, with the sole exception of an NRB occurrence in the NM group. No epileptic seizures were recorded.

All 115 exams revealed abnormal findings. Across the entire sample, the VPSG study provided positive determinant findings that confirmed the primary prestudy hypothesis in 50 cases (43.5%) and supportive findings that would corroborate the primary hypothesis when combined with clinical information in another 53 cases (46.1%). At the time the VPSG study was performed, there was a prestudy suspicion of group overlap in 69 cases (60.0%) in the entire sample. Therefore, most VPSG findings eventually supported some aspects of the prestudy hypotheses.

### Diagnostic Yield of the VPSG with Extended EEG Protocol

In total, 13 exams (11.3%) were acquired using the extended EEG-PSG protocol. Most of these cases ( *n* = 8; 61.5%) involved suspected OSA in the context of epilepsy. Two (15.4%) patients had a history of stroke accompanied by abnormal EEG findings, but no seizures. The reasons for selecting an extended EEG montage were unclear in 3 (23.1%) exams: 2 concerned suspected OSA and 1, suspected RBD.

Interictal epileptiform EEG activity was observed in 10 out of 13 (76.9%) recordings. In every instance, these findings were consistent with previous daytime sleep EEG results. The video recordings revealed additional findings in 2 (15.4%) studies, 1 involving DEB and another featuring NRB along with waking up with leg discomfort. The final reported diagnoses included mild to moderate OSA in 9 cases (69.2%) and definite or prodromal RBD in 2 cases (15.4%).

## Discussion

The current study reviewed data from VPSGs conducted over 2 years in a public university-affiliated hospital in Porto Alegre. The main referrals were patients with a movement disorder related to parkinsonism and/or tremor, epilepsy, neuromuscular and cerebrovascular disease, most frequently referred due to suspected OSA and/or RBD. Nocturnal motor behaviors, refractory insomnia or other sleep-disorder symptoms deemed to require VPSG rather than a standard respiratory sleep study were included. There was a high prevalence of psychoactive drug use, particularly antidepressants, along with other medications that may influence sleep architecture or cause sleep-related adverse effects. 25 The procedure was highly effective, yielding positive or supportive findings for the primary prestudy hypothesis in 103 (89.6%) of the cases.

In the NM group, the occurrence of abnormal behaviors was inconspicuous and significantly lower than in the M group, suggesting that the prestudy clinical evaluations were effective in identifying nocturnal motor behaviors. However, RWA was present in 7 (13.0%) of those records. Notably, none of these cases corresponded to isolated RWA, a condition likely indicative of an early stage of iRBD, considered itself a prodromal form of Alpha-synucleinopathy. 26 All 7 (13.0%) cases could be explained by the presence of some form of neurodegenerative disease and/or antidepressant use. This underscores the importance of conducting standard sleep studies with video information and paying special attention to excessive motor activity during REM sleep, as currently recommended by international guidelines and the ABS. 11 12 In the presence of excessive motor activity, human confidence for scoring REM sleep heavily relies on video data. 6 Conversely, there was a high prevalence of sleep-disordered breathing in the M group, particularly among patients with a prestudy suspicion of RBD. For this subgroup, the likelihood of having moderate-to-severe OSA was nearly as high as that of receiving a positive RBD diagnosis. Moreover, the occurrence of OSA of any severity exceeded the combination of positive and incomplete findings for RBD. This result aligns with our expectations, as a high prevalence of sleep-disordered breathing is commonly reported in association with synucleinopathies and within the broader context of nocturnal motor behaviors. 27 28 However, the present retrospective study lacked sensitivity to determine whether the referring physicians were fully aware of this significant comorbidity. Questionnaires administered within the laboratory identified snoring and witnessed apnea in most of these patients; however, such information was frequently absent from the records.

Indirect evidence suggests that the real-world management of suspected RBD may not adhere to standard international guidelines. A recent study from the United States, 29 for instance, revealed that, in that high-income country, many RBD diagnoses are still established without PSG, or without mentioning RWA in the PSG reports. In fact, the 2022 guidelines by the International RBD Study Group have been regarded by World Sleep Society specialists as difficult to implement in most health systems and medical communities across member countries. 30 Considering, for instance, the current VPSG availability, the estimated RBD prevalence of 1% among the elderly and the estimated elderly population of 2.2 million in Rio Grande do Sul, it would take us more than 300 years to comprehensively investigate RBD under present-day conditions. 16 31 The strongly-restrained demand for this examination poses a paradox for the clinicians who need to diagnose and treat RBD in similar conditions. Sleep-disordered breathing is a primary differential diagnosis for RBD, with significant overlap, particularly in the context of Alpha-synucleinopathies. 26 32 Respiratory events can interfere with RWA scoring. 33 The current guidelines caution against RWA scoring if REM AHI is ≥ 15. 6 Screening for OSA in this population is challenging, since standard OSA screening questionnaires lose effectiveness. 34 Home type-2 studies are not recommended for significant non-respiratory sleep disorders such as parasomnia. 35 Thus, performing type-1 PSG to rule out or treat OSA before the empirical treatment for RBD may be considered, acknowledging that this is not the gold standard for RBD diagnosis, and keeping in mind that a definite RBD diagnosis has important prognostic and medicolegal implications, and should always be pursued in ideal conditions.

The small number of VPSG-EEG studies precludes definitive statements about the diagnostic yield of this procedure. However, compared with previous exams, no new EEG findings were identified. Given that VPSG-EEGs were primarily conducted to investigate suspected OSA in the context of an existing epilepsy diagnosis, ASM was maintained, which likely contributed to the absence of recorded seizures. 2 36 Overall, out of 14 cases, the extended EEG protocol may have only been beneficial for 1 (7.1%) patient. Further research is needed to determine the cost-effectiveness of this procedure in our region, as our results do not justify one-night, outpatient VPSG with extended EEG for seizure confirmation.

The current study has several limitations. The sample was collected between 2013 and 2015. Therefore, it did not benefit from guidelines pertaining to PSG acquisition, sleep-scoring rules and sleep-disorder diagnosis which were issued in the past 10 years. This could have an impact on the accuracy of our results, particularly concerning the lack of quantitative RWA scoring. The OSA diagnosis was based on scoring criteria that did not account for milder events such as respiratory effort-related arousals, making it less sensitive compared with current guidelines. Only VPSG studies were included, so no direct comparison between VPSG and type-1 PSG was performed. Additionally, activation methods such as sleep deprivation and deep-sleep interruption, which could have enhanced sensitivity for NREM arousal parasomnias, were not employed. 37 Nevertheless, the results of the present retrospective study objectively corroborate current national and international recommendations for standard (type-1) PSG studies to include synchronized video acquisition. 5 6 7 11 12 A significant strength of the current study is its focus on a population sourced outside from mainstream sleep facilities, which can aid healthcare authorities in developing tailored practice protocols. 38

The development of sleep medicine in Latin America, home to 660 million people, is highly uneven. Most Latin American countries lack formal training in sleep medicine. Only a few countries like Argentina, Uruguay, Colombia, and Brazil have healthcare systems that offer PSG services. 39 In Brazil, few studies have systematically described VPSG procedures. One study 40 from Southern Brazil examined VPSG findings in a sample of 17 patients with spinocerebellar ataxia type 2 (SCA2), identifying significantly-reduced REM sleep time and REM density, but no excessive REM sleep motor activity in these patients. All other studies 41 42 43 have been conducted in the major referral center of São Paulo, focusing on PD. One study 41 investigated the prevalence of excessive fragmentary myoclonus (EFM) in a cohort of 59 PD patients and found it to be of 62.7%. Obstructive sleep apnea was present in 72.9% and 40.9% of the patients with and without EFM respectively. Another study 42 assessed the accuracy of clinical interviews in detecting RBD among 88 PD patients, reporting high sensitivity (87.5%) and low specificity (42.1%) for the clinical interviews. Among their 55 patients with VPSG-confirmed RBD and 11 patients with VPSG unconfirmed RBD, OSA prevalence was of 63.6% and 72.7% respectively. Of note, RBD diagnosis could not be verified for 18.1% of that sample due to insufficient REM sleep time. A third study 43 validated the Brazilian version of the RBD Screening Questionnaire (RBDSQ_Br) in a cohort of 69 PD patients, confirming its validity and reliability. In that cohort, 68.1% of the patients had OSA, while 72.5% had RBD. These studies primarily involved patients with an established PD diagnosis; thus, further research is needed to explore local referral patterns for suspected iRBD. The population in the current study was more heterogeneous, as only 26 (48.1%) of the patients with sleep-related motor symptoms had an established movement disorder with tremor and/or parkinsonism; nevertheless, the sample size was insufficient to specifically investigate iRBD. Overall, these findings indicate significantly limited access to VPSG and in-laboratory RBD diagnosis in this world region.

## Conclusion

In a world region with limited VPSG resources, our results show that the single most prevalent and important reason for VPSG referral was the investigation of RBD versus OSA diagnoses. However, most patients referred to VPSG had prior neurological diagnoses and were investigating OSA in the absence of motor complaints, suggesting they might benefit from referral to type-1 PSG, which is more readily available. Additionally, suspected RBD cases were more likely to be diagnosed with sleep apnea than with RBD, and less than half of the cases of RBD suspicion were confirmed, suggesting screening accuracy may be improved, particularly through more thorough history taking. Finally, most RBD diagnoses were established for patients who already had a neurodegenerative disorder, suggesting the protracted stage during which iRBD precedes phenoconversion to Alpha-synucleinopathy, which warrants special clinical attention, is largely being missed.

In world regions where VPSG is scarce, clinicians must recognize the high probability of sleep apnea among patients referred for motor activity during sleep. When RBD is suspected and VPSG is unavailable, even for patients with PD, we recommend performing type-1, 'respiratory' PSG. This approach enables clinicians to investigate OSA rather than initiate an empirical, possibly-inappropriate treatment for RBD. As the demand for precise quantification of motor activity during REM sleep continues to increase, it is crucial to enable access to VPSG for a broader spectrum of patients, including early iRBD cases and those patients with other forms of movement disorders.
